# Fire Detection in Ship Engine Rooms Based on Deep Learning

**DOI:** 10.3390/s23146552

**Published:** 2023-07-20

**Authors:** Jinting Zhu, Jundong Zhang, Yongkang Wang, Yuequn Ge, Ziwei Zhang, Shihan Zhang

**Affiliations:** College of Marine Engineering, Dalian Maritime University, Dalian 116026, China; zjt123@dlmu.edu.cn (J.Z.);

**Keywords:** fire detection, deep learning, YOLOv7-tiny, coordinate attention mechanism, partial convolution, SIoU

## Abstract

Ship fires are one of the main factors that endanger the safety of ships; because the ship is far away from land, the fire can be difficult to extinguish and could often cause huge losses. The engine room has many pieces of equipment and is the principal place of fire; however, due to its complex internal environment, it can bring many difficulties to the task of fire detection. The traditional detection methods have their own limitations, but fire detection using deep learning technology has the characteristics of high detection speed and accuracy. In this paper, we improve the YOLOv7-tiny model to enhance its detection performance. Firstly, partial convolution (PConv) and coordinate attention (CA) mechanisms are introduced into the model to improve its detection speed and feature extraction ability. Then, SIoU is used as a loss function to accelerate the model’s convergence and improve accuracy. Finally, the experimental results on the dataset of the ship engine room fire made by us shows that the mAP@0.5 of the improved model is increased by 2.6%, and the speed is increased by 10 fps, which can meet the needs of engine room fire detection.

## 1. Introduction

Ship fires are one of the main problems that endanger the safety of ships. Since ships are sailing far away from land for a long time at sea, the fire risk factor is high and difficult to deal with, which often causes incalculable losses. For example, a massive fire and explosion accident on the 4400 teu Hanjin Pennsylvania in 2002 caused nearly USD 100 million in damages to the insurance company. In July 2012, a container ship, the 6732 teu MSC Flaminia, caught fire in the Atlantic Ocean and killed three sailors. In 2018, a ship fire accident aboard the Maersk Honam in the Indian Ocean generated the largest container ship general average claim in shipping history, with the final insurance payout expected to exceed USD 500 million. In January 2019, a 7150 teu container ship called Yantian Express was also involved in a major fire accident in the Atlantic Ocean. According to the analysis of the Ship Safety Risk Report 2022, the shipping industry has shown a more positive safety trend over the past decade, but as ships age, fires on board are on the rise. In the past five years, fires and explosions have overtaken shipwrecks and collisions as the number one cause of Marine insurance losses. With the rapid development of ports and shipping, ships are increasingly large, environmentally friendly, professional, and intelligent, and the overall safety of ships has been widely improved. However, the statistics do not show a significant improvement in ship fire accidents.

The engine room of a ship is the core compartment that provides power to the ship and guarantees the normal operation of the ship. However, due to the complex structure and combustible materials inside the cabin, 75% of all ship fires start in the engine room, and nearly two-thirds of engine room fires occur in the main and auxiliary engines, or their related components, such as the turbochargers. Therefore, engine room fire detection is extremely important. A method that can quickly and accurately detect an engine room fire on ships is important to reduce the damage to people and property caused by ship accidents and can play a positive role in further improving ship damage control systems and improving ship fire prevention and control technology.

Traditional fire detection technology is generally judged by collecting data from various types of indoor sensors. When the detected parameter values reach the threshold of the sensor settings, an alert will be issued. Early sensor technology was mainly based on the “point sensor” of particle activation, based on heat, gas, flames, smoke, and other important fire features [1]. Depending on the sensor detection objects, they can be divided into smoke sensors, temperature sensors, photosensitive sensors, and special gas sensors. These detectors can detect fires from multiple angles, but there are also defects with limited detection range, accuracy, and response speed at the same time. The particles must pass through a certain distance to reach the sensor; for example, for smoke detectors often installed on a building ceiling, only a small share of smoke occurs when a fire has just occurred, which is not enough to trigger the fire alarm. The alarm will be triggered only when the smoke reaches a certain concentration and rises up to the sensor. This time difference may cause the fire to spread quickly to the point where it cannot be controlled. Simply put, if a fire is far from the sensor, it may not be detected immediately [2]. In addition, the sensor is also easily affected by factors such as light and dust, causing potential misjudgments. In fact, both flames and smoke have certain static and dynamic characteristics, such as color and movement. Point sensors do not use these important features to detect fires [3]. It can be seen that traditional fire detectors have their own advantages, but their disadvantages are also obvious. Almost all of the above fire detectors could be limited by the environment while having multiple sensors in place will increase the cost and difficulty of placement.

In recent years, with the rapid development of computer vision, image processing technology, the gradual improvement of hardware computing capabilities, and the popularization of video surveillance networks, people have gradually shifted to focus on the development of fire detection technology to detect fires. Video fire testing based on deep learning has become a popular research field, with its characteristics of fast response and high accuracy. Algorithms based on convolutional neural networks can extract deeper image features, suitable for fire detection in complex venues such as engine rooms. On the one hand, modern ships are becoming more and more intelligent and automated, and video surveillance systems are becoming increasingly mature, which provides the possibility of using monitoring and deep learning technology for fire detection in the engine room. On the other hand, video-oriented fire detection has been successfully applied in many fields, it can detect indoor scenes, such as office fires, and can also detect outdoor scenes, such as forest fires. This lays a foundation for its application in ships. Fire detection technology based on deep learning mainly inputs the engine room scene through real-time video surveillance. Compared with traditional sensor detection technology, it has the following advantages:

1. It has a wide detection range and fast reaction speed. Fire detection technology based on deep learning can respond to a fire at its early stage;
2. It can track and predict the flames and evaluate their development trend. At the same time, it will pave the way for the directional fire extinguishing of the unmanned engine room of a future ship;
3. Compared with the traditional fire detector, which can only provide limited information, it can record the fire process completely, and contribute to the subsequent accident investigation and future safety regulations;
4. It is convenient to arrange. Only a few surveillance cameras are needed to monitor the entire engine room;
5. It has low environmental requirements and can also be compatible with other types of fire detectors.

In summary, the fire detection technology based on deep learning has obvious advantages, and it has very important research significance and practical application value in ensuring the safety of navigation and improving the automation and intelligence of the cabin.

Although fire detection based on deep learning has developed rapidly, this method is still in the initial stage in its application and research of the ship engine room. The main problems are as follows:

1. There are too little image data of cabin fires. Deep learning is an algorithm based on big data. The more samples used in model training, the more features are learned in models, which makes the model more effective. However, there are currently no public data sets related to ship cabin fires, and it is difficult to obtain ship engine room fire image data, so we cannot build a large-scale cabin fire data set;
2. The complex environment inside the engine room of the ship may affect the performance of the algorithm in the actual scene. A model may perform well in training and averagely well in testing. In particular, the space of the engine room is large and it has a lot of equipment, complex pipelines, and many red or yellow signs, which are similar to the color of the fire. If the robustness of the algorithm is not high, the detection and location of the fire could be affected.

In response to the above problems, this article proposes a fire detection algorithm based on the improved YOLOv7-tiny [4]. The specific work is as follows:

1. We used the 3D virtual engine room simulator of Dalian Maritime University to collect engine room fire image data. Combined with real fire images in other scenarios, we construct a ship engine room fire dataset for training models.
2. We improve the original YOLOv7-tiny algorithm. More specifically, partial convolution (PConv) is used to replace part of the regular convolution module in the original network to reduce redundancy in the calculation process. Then the coordinate attention (CA) mechanism is added to strengthen the feature extraction ability of the model to increase the robustness of the model. Finally, the SCYLLA-IoU (SIoU) loss function is used to accelerate the efficiency of training and improve the accuracy of inference.

The remaining parts are arranged as follows: the second part discusses related work; the third part introduces the improvement method; the fourth part conducts experimental verification; the fifth part summarizes and prospects the research.

## 2. Related Work

The technology of fire detection using vision can be mainly divided into traditional detection methods based on image processing and detection methods based on deep learning. Fire recognition based on image processing technology mainly obtains the fire area by analyzing and extracting the dynamic and static characteristics of the flames, such as color features, shapes, and other appearance features; then, the extracted features are passed into the machine learning algorithm for recognition. Chen et al. [5] proposed an early fire warning method based on video processing. The basic idea is to extract fire pixels and smoke pixels using chromaticity and disorder measurements based on the RGB (red, green, blue) model. Wu et al. [6] proposed a dynamic fire detection algorithm for surveillance video based on the combination of radiation domain feature models. Foggia et al. [7] proposed a method to detect fires by analyzing videos captured by surveillance cameras, which combined the complementary information of color, shape change, and motion analysis through multiple expert systems. Arthur K et al. [8] conducted research on video flame segmentation and recognition; in this paper, the Otsu multi-threshold algorithm and Rayleigh distribution analysis method were used to segment the image to obtain an accurate and clear flame image. Then the flame image was identified by the method of combining the nearest neighbor algorithm with the flame centroid feature. S. R. Vijayalakshmi et al. [9] proposed a method integrating color, space, time, and motion information to locate fire areas in video frames. The method fuses the characteristics of the smoke with the burning flame to remove the false fire area. Binti Zaidi et al. [10] developed a fire identification system based on the consistency rules of R, G, B, Y, Cb, and Cr component values in images. Wang et al. [11] combined the dynamic and static characteristics of the flame in the video for fire detection, effectively reducing the impact of environmental factors on the results. Although researchers have conducted many studies of smoke and flame images, they have found only a few simple image features, which are not enough to cover the complex fire types and scenarios.

In recent years, with the rapid development of deep learning, it has been successfully applied to many fields, such as image classification, object detection, speech recognition, natural language processing, and so on. Its applications range from simple classification of cats and dogs to complex medicine. For example, Yuan et al. [12] used convolutional neural networks (CNN) and bidirectional long short-term memory (BiLSTM) to predict anticancer peptides (ACPs). Shervan Fekri-Ershad et al. [13] proposed a multi-layer perceptron (MLP) neural network with deep features to analyze cell images. The fire detection method based on convolutional neural network (CNN) has also been widely used, which has significant potential advantages, such as fast response speed, wide detection range, high precision, and low detection cost. At present, there are many excellent target detection models, and the first-stage algorithms include OverFeat [14], Single Shot MultiBox Detector (SSD) [15], R-SSD [16], YOLO (you only look once) series [4,17,18,19,20], etc. The two-stage algorithms include R-CNN [21], Fast R-CNN [22], Faster R-CNN [23], Mask R-CNN [24], and so on. Barmpoutis et al. [25] used the Faster R-CNN model to identify fires, achieving high detection accuracy, but the speed was not fast enough. Shen et al. [26] used the YOLOv1 model to achieve flame detection, but there is still much room for improvement. Qian [27] et al. introduced channel-wise pruning technology to reduce the number of parameters in YOLOv3, making it more suitable for fire monitoring systems. Wang et al. [28] proposed a lightweight detector, Light-YOLOv4, which considers the balance between performance and efficiency and has good detection performance and speed in embedded scenarios. Wu [29] et al. improved the SPP module and activation function of YOLOv5 to improve the robustness and reliability of fire detection. Xue [30] et al. introduced the convolutional block attention module (CBAM) and bidirectional feature pyramid network (BiFPN) into YOLOv5, which improved the detection of small targets in forest fires. The application of fire detection based on deep learning technology in ships is still in the initial stage. Wang et al. [2] proposed a video-based ship flame and smoke detection method to overcome the shortcomings of traditional fire detection equipment. First, using the fuzzy C-means clustering algorithm to create the dominant flame color lookup table (DFCLT), then, the changed region in the video frame is extracted and the alarm is triggered by the contrast of the pixels. Park et al. [31] further tested the performance of Tiny-YOLOv2 for fire detection in the environment of the ship engine room. Wu et al. [32] proposed an improved YOLOv4-tiny algorithm for accurate and efficient ship fire detection. They improved the detection accuracy of small target objects by adding a detection layer and Squeeze-and-Excitation attention (SE) module to the network. In general, detection methods based on deep learning technology can not only automatically extract image details and features but also learn deeper features of the targets so that it has a better feature extraction effect than traditional image processing technology.

## 3. Methods

This section elaborates on the network structure of YOLOv7-tiny and the improvement methods, including improving the backbone network, adding the attention mechanism, and modifying the loss function.

### 3.1. The Model Structure of YOLOv7-Tiny Network

**Figure 1:**
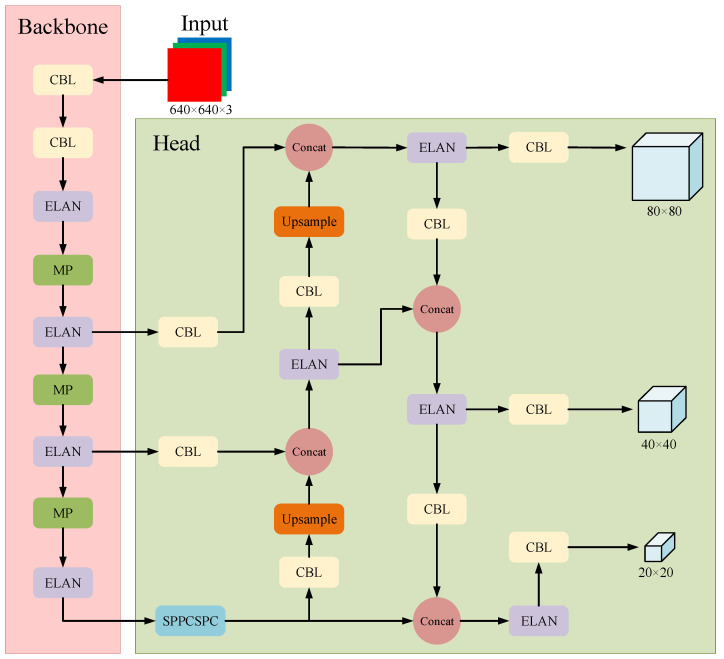
The structure of the YOLOv7-tiny network.

Created in 2022, YOLOv7 [3] is a relatively new work in the YOLO series. It is a kind of target detection network with high speed and precision, easy training, and deployment, and it surpasses previous object detectors in both speed and accuracy, ranging from 5 FPS to 160 FPS. In order to better meet the requirements of real-time fire detection, the YOLOv7-tiny model with the smallest amount of computation and parameters is chosen as the baseline model in this paper. Figure 1 shows the structure of YOLOv7-tiny.

According to the structure diagram, the YOLOv7-tiny network consists of an input layer, backbone network, and head network. The input part uses techniques such as adaptive image scaling and Mosaic data augmentation. The image size is uniformly converted to 640 × 640 × 3. Mosaic data enhancement is to stitch together four pictures by random scaling, cropping, arrangement, and other processing and can increase the number of targets, enrich the diversity of data, improve the robustness of the network, and the ability to detect small objects.

**Figure 2:**
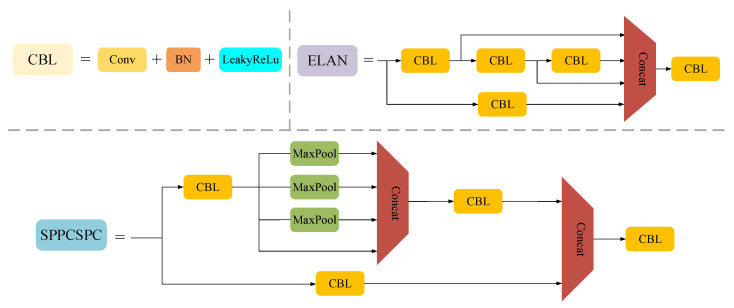
Structure of each module.

In the backbone network, the picture goes through a series of CBL modules, ELAN modules, and MP modules to reduce the length and width, and increase the number of channels. The module structure is shown in Figure 2. The CBL module represents a convolutional layer, a BN layer (batch normalization), and an activation function. Unlike YOLOv5, YOLOv7-tiny uses LeakyReLU as the activation function, which evolved from the ReLU (rectified linear unit) activation function. Compared with ReLU, LeakyReLU handles negative values better and the overall function interval is not zero, which solves the problem that part of the network parameters cannot be updated. The formula is as follows:

(1) $$\;\mathrm{LeakyReLU}\;(x)=\left\{\begin{array}{cc} x, & \;\mathrm{if}\;x\geq 0\\ \alpha x, & \;\mathrm{otherwise}\; \end{array}\right.$$

ELAN module is an efficient network architecture that enables the network to learn more features and be more robust by controlling the shortest and longest gradient paths. It has two branches, one of which goes through convolution to change the number of channels. The other uses multiple convolution for feature extraction. Finally, the four features are superimposed together to extract the result. The image is extracted in the backbone network and then fused in the head network. The fusion part of YOLOv7-tiny is similar to that of YOLOv5, using the PANet structure. The SPPCSPC module in the head network can obtain multi-scale target information while keeping the size of the feature map unchanged. The function of SPP is to increase the receptive field. It obtains different receptive fields through maximum pooling so that the algorithm can adapt to different-resolution images. According to the structure diagram, four different Maxpools represent that it can handle different objects and can be used to distinguish between large and small targets. The CSP structure first divides the features into two parts, one of which is processed conventionally, and the other part is processed with the SPP structure. Finally, the two parts are merged together.

YOLOv7-tiny mainly calculates three loss functions, namely, the bounding box loss, the classification loss, and the target confidence loss. BCEWithLogitsLoss (binary cross entropy loss with log) is used for target confidence loss and classification loss, and the CIOU loss is used to calculate the box loss. During the post-processing of the target detection phase, YOLOv7-tiny uses Non-Maximum Suppression (NMS) to filter multiple target candidate boxes and eliminate redundant candidate boxes, which ensures that the algorithm only gets one detection box for each object in the end.

### 3.2. Partial Convolution

**Figure 3:**
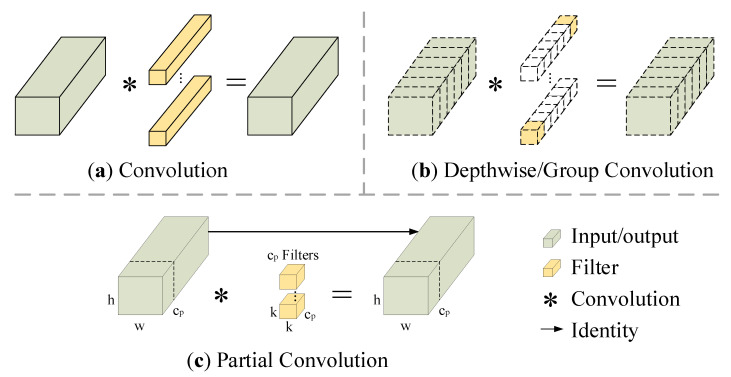
Three different convolution modes. (**a**) Regular convolution. (**b**) Depthwise convolution (DWConv). (**c**) Partial convolution (PConv).

Whether on land or on board, real-time fire detection is very important. A fast-operating network model can detect fires earlier, greatly reducing the harm they cause. Many researchers are working on designing fast neural networks. Rather than requiring more expensive equipment, they tend to design fast neural networks that are cost-effective. So a lot of work has been carried out on how to reduce computational complexity. Computational complexity is measured by the number of floating-point operations (FLOPs). MobileNet [33], ShuffleNet [34], and GhostNet [35] utilize depthwise convolution (DWConv) or group convolution (GConv) to extract features, and these networks all have low FLOPs. However, Chen et al. [36] found that they are not actually fast enough because the operators often suffer from the effect of increased memory access in the process of reducing FLOPs. Therefore, a new partial convolution (PConv) has been proposed to extract spatial features more effectively by reducing redundant computation and memory access at the same time. Various convolution forms are shown in Figure 3.

**Figure 4:**
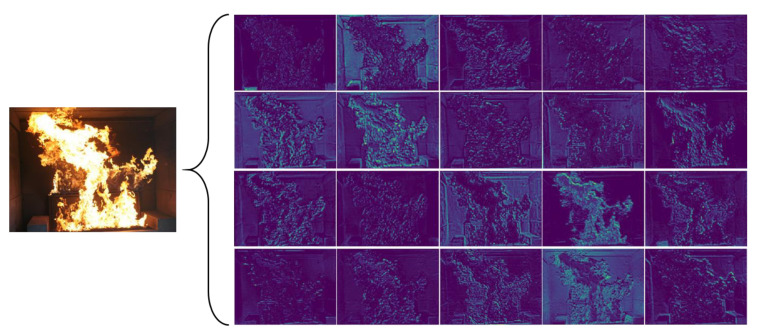
Visualization of the feature maps.

As shown in Figure 4, the feature maps are highly similar between different channels, which brings about computational redundancy. Figure 3c shows how PConv works. To reduce computational redundancy, it simply applies regular convolution on part of the input channel for feature extraction and keeps the rest of the channels unchanged. For the regular convolution and the depthwise convolution, they have FLOPs *h* × *w* × *k*^2^ × *c*^2^ and *h* × *w* × *k*^2^ × *c*, respectively, and the FLOPs of the partial convolution are

(2) $$h\times w\times k^{2}\times c_{p}^{2}$$

When *c_p_*/*c* = 1/4, the FLOPs of the partial convolution is 1/16 of the regular convolution. Thus PConv achieves lower FLOPs than regular convolution and higher FLOPs than depthwise convolution.

**Figure 5:**
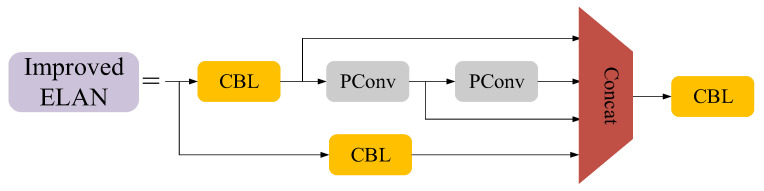
Improved ELAN module.

In this paper, we combine the ELAN module in the backbone network with PConv to extract features more efficiently. As shown in Figure 5, we replaced some CBL modules in the ELAN module with PConv.

### 3.3. Coordinate Attention (CA) Mechanism

**Figure 6:**
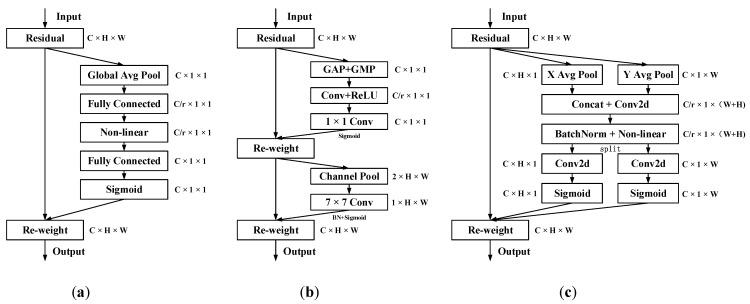
Structure of the attention mechanisms. (**a**) squeeze-and-excitation attention (SE). (**b**) convolutional block attention modules (CBAM). (**c**) coordinate attention (CA) mechanism.

The attention mechanism is a concept proposed to mimic the human nervous system. It has been widely used in various fields of deep learning in recent years and has shown great success in tasks of image segmentation, speech recognition, and natural language processing. Because of bottlenecks in information processing, humans selectively focus on some information while ignoring others. Similarly, when a neural network processes a large quantity of input information, it quickly focuses on some of the key information for processing, which is the attention mechanism. Its essence is to enhance the useful feature information and suppress the useless information to improve detection accuracy. The attention mechanism can be generally divided into a channel attention mechanism and a spatial attention mechanism, such as the squeeze-and-excitation attention (SE) [37] and convolutional block attention modules (CBAM) [38], etc. The SE module only considers the information between channels while ignoring the location information. Although CBAM is improved, it still lacks the ability of long-distance relation extraction. In contrast, the coordinate attention (CA) mechanism [39] not only obtains inter-channel information, but also considers orientation-related location information, which helps the model to better locate and identify the target. In addition, it is flexible and lightweight enough to easily plug into the core modules of mobile networks. Due to the complexity and variability of the engine room environment, the model needs to improve its ability to express the flame’s characteristics. The two advantages of coordinate attention mentioned above is greatly assist our model. Therefore, we added the attention mechanism to the backbone network. The structure of the three attention mechanisms is shown in Figure 6, where (a) is SE, (b) is CBAM, and (c) is the coordinate attention (CA) mechanism.

Different from SE in Figure 6a and CBAM in (b), in order to avoid losing spatial information of objects, CA does not directly use global max pooling and global average pooling, but is composed of two similar parallel stages. The location information is embedded into the channel attention by processing the high and wide direction features.

The specific operation is to first pool the input feature graphs of size C × H × W in the X direction and the Y direction, respectively, to generate the feature graphs of size C × H × 1 and C × 1 × W. The calculation formula is as follows:

(3) $$z_{c}^{h}(h)=\frac{1}{w}\sum_{0\leq i<\omega }x_{c}(h,i)$$

(4) $$z_{c}^{w}(w)=\frac{1}{H}\sum_{0\leq j<H}x_{c}(j,w)$$

where $z_{c}^{h}(h)$ is the output of the channel whose number is *c* and height is *h*, and $z_{c}^{w}(w)$ is the output of the channel whose number is c and width is *w*.

Secondly, the width and height feature maps of the global receptive field are spliced together and passed to the 1 × 1 convolution module to reduce the dimension to the original C/r. After batch normalization, the feature graph F1 is fed into the nonlinear activation function to obtain the feature maps in the form of 1 × (W + H) × C/r. The calculation formula is as follows:

(5) $$f=\delta \left(F_{1}\left(\left[z^{h},z^{w}\right]\right)\right)$$

where *F*_1_ is a 1 × 1 convolution transformation, [·,·] is a concatenation operation, and *δ* is a nonlinear activation function.

Then, the feature map is decomposed into two independent tensors $f^{h}$ and $f^{w}$, and two 1 × 1 convolutions are used to obtain feature map $F_{h}$ and $F_{w}$ with the same number of channels as the input x. The Sigmoid activation function is used to obtain the attention weights in the height direction and the width direction of the feature graph. The calculation formula is as follows:

(6) $$g^{h}=\sigma \left(F_{h}\left(f^{h}\right)\right)$$

(7) $$g^{w}=\sigma \left(F_{w}\left(f^{w}\right)\right)$$

where $F_{h}$ and $F_{w}$ are 1 × 1 convolution transformations and *σ* is the sigmoid function.

Finally, the original feature map is weighted by multiplication, and the output *y* of the CA attention module is calculated. The formula is as follows:

(8) $$y_{c}(i,j)=x_{c}(i,j)\times g_{c}^{h}(i)\times g_{c}^{w}(j)$$

### 3.4. SIoU Loss

The loss function is crucial in the process of model training because it can calculate the gap between the model and the actual data, and determine how well the model performs. The proper loss function is helpful to make the model converge faster and obtain better results in the training process. Traditional losses such as GIoU [40], DIoU [41], and CIoU [41] only consider the distance, overlap area, and aspect ratio between the prediction box and the ground truth but do not consider the angle between the real box and the prediction box, which leads to a slow convergence speed. In order to solve the above problems, Gevorgyan proposed the SCYLLA-IoU (SIoU) loss function [42]. The SIoU loss function redefines the penalty measure by considering the vector Angle between the required regressions. This consideration can greatly speed up the training convergence process. It predicts that the box will first move to the nearest axis (x or y). Then the prediction box is regressed along this axis. SIOU consists of four parts:

1. Angle cost

**Figure 7:**
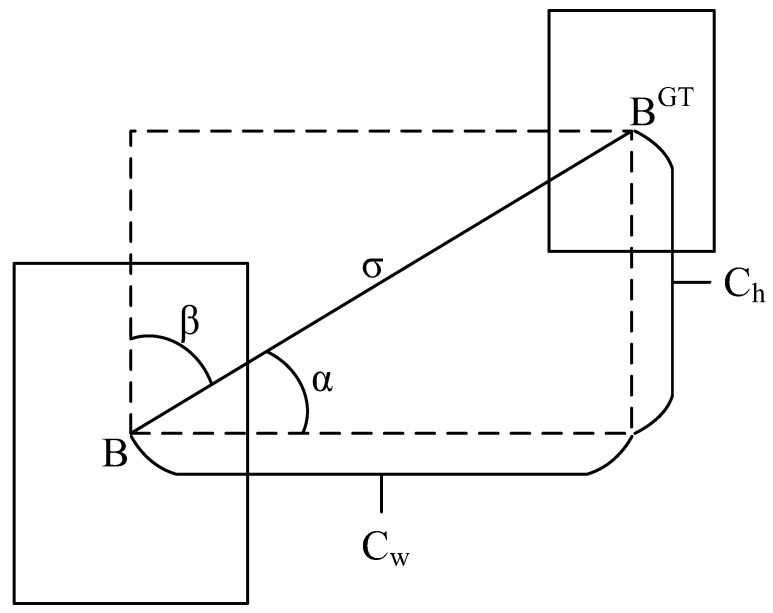
Calculation of angle cost.

The angle cost diagram is shown in Figure 7, where B and B^GT^ are the prediction box and the ground truth box, and *α* and *β* are the angles to the horizontal and vertical directions, respectively. $c_{h}$ is the height difference between the center points of the prediction box and the ground truth box. σ is the distance between the center point of the prediction box and the ground truth box.

The regression direction of the prediction box is determined by the magnitude of the angle. To achieve this, the following strategy is used to optimize the angle parameter θ:

(9) $$\theta =\left\{\begin{array}{l} \alpha ,\;\alpha \leq \pi /4\\ \pi /2-\beta ,\;\mathrm{else}\; \end{array}\right.$$

If *α* ≤ π/4, the convergence process will first minimize *α*, otherwise minimize *β*.

The angle cost is calculated as follows:

(10) $$\Lambda =1-2\times \sin^{2}\left(\arcsin\left(\frac{c_{h}}{\sigma }\right)-\frac{\pi }{4}\right)=\cos\left(2\times \left(\arcsin\left(\frac{c_{h}}{\sigma }\right)-\frac{\pi }{4}\right)\right)$$

(11) $$\frac{c_{h}}{\sigma }=\sin(\alpha )$$

(12) $$\sigma =\sqrt{{\left(b_{c_{x}}^{gt}-b_{c_{x}}\right)}^{2}+{\left(b_{c_{y}}^{gt}-b_{c_{y}}\right)}^{2}}$$

(13) $$c_{h}=\max\left(b_{c_{y}}^{gt},b_{c_{y}}\right)-\min\left(b_{c_{y}}^{gt},b_{c_{y}}\right)$$

2. Distance cost

**Figure 8:**
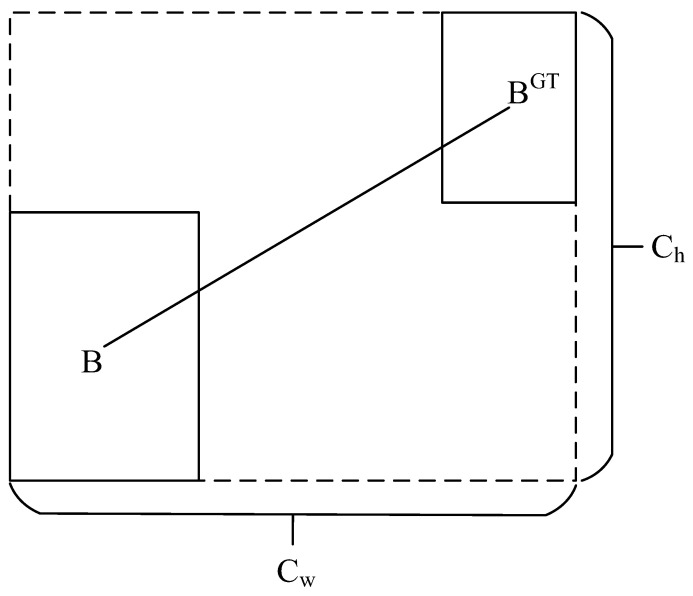
Calculation of distance cost.

The distance cost diagram is shown in Figure 8.

The distance cost is redefined according to the angle cost, and its calculation formula is as follows:

(14) $$\Delta =\sum_{t=x,y}\left(1-e^{-\gamma \rho_{t}}\right)=2-e^{-\gamma \rho_{x}}-e^{-\gamma \rho_{y}}$$

where $\rho_{x}={\left(\frac{b_{c_{x}}^{gt}-b_{c_{x}}}{c_{w}}\right)}^{2}$, $\rho_{y}={\left(\frac{b_{c_{y}}^{gt}-b_{c_{y}}}{c_{h}}\right)}^{2}$, γ=2−Λ.

3. Shape cost

The shape cost is calculated as follows:

(15) $$\Omega =\sum_{t=w,h}{\left(1-e^{-wt}\right)}^{\theta }={\left(1-e^{-w_{w}}\right)}^{\theta }+{\left(1-e^{-w_{h}}\right)}^{\theta }$$

where $w_{w}=\frac{\left|w-w^{gt}\right|}{\max\left(w,w^{gt}\right)}$, $w_{h}=\frac{\left|h-h^{\mathrm{gt}}\right|}{\max\left(h,h^{\mathrm{gt}}\right)}$.

4. IoU cost

**Figure 9:**
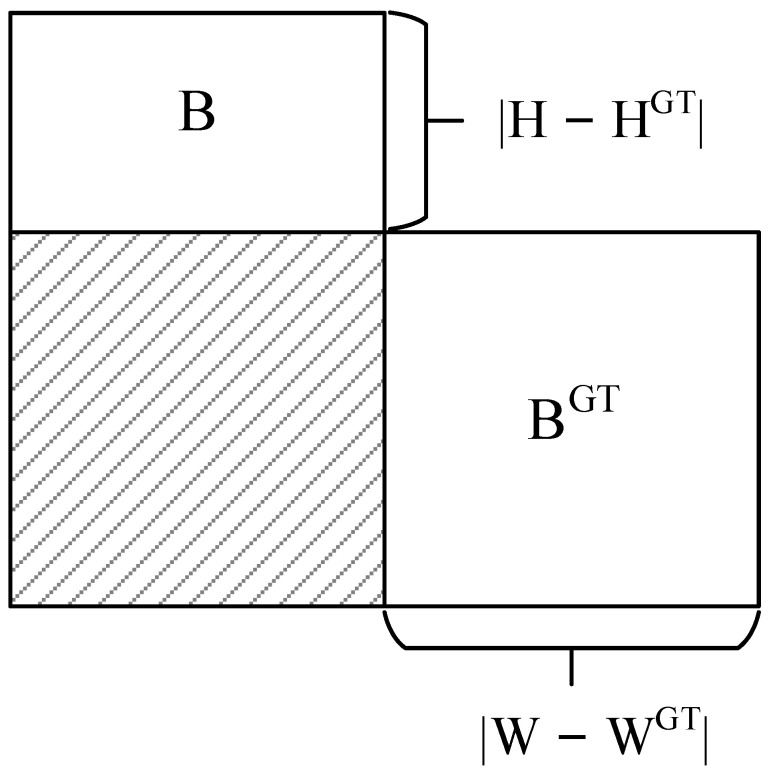
IoU diagram.

The IoU cost is shown in Figure 9, where

(16) $$\mathrm{IOU}\left(B,B_{gt}\right)=\left|\frac{B\cup B_{gt}}{B\cap B_{gt}}\right|$$

In summary, the SIoU loss function is defined as follows:

(17) $${\mathrm{Loss}}_{\mathrm{SIoU}}=1-\mathrm{IoU}+\frac{\Delta +\Omega }{2}$$

## 4. Experiments

**Figure 10:**
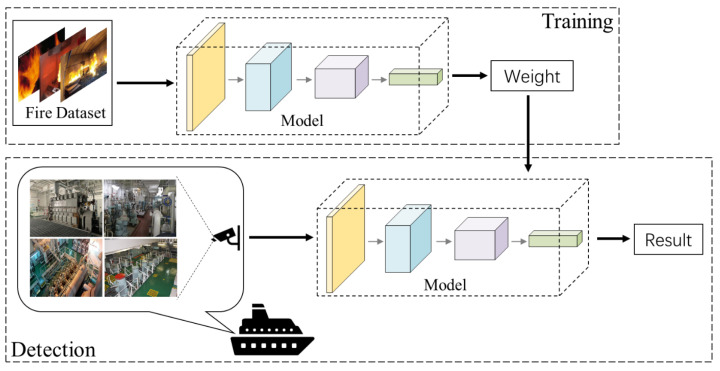
The process of fire detection in an engine room.

The process of fire detection in an engine room is shown in Figure 10. In the training part, the prepared dataset is loaded into the model for training, and then the weight is generated. In the engine room of the real ship, surveillance cameras are used to monitor the engine room, and the results are generated after the surveillance video is fed into the model with weighting.

### 4.1. Dataset

**Figure 11:**
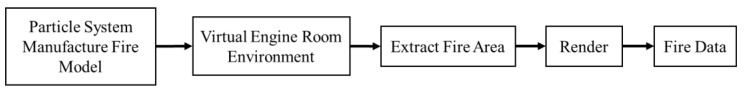
Process of simulated data production.

The training of the model requires a large amount of data. However, it is often difficult to obtain engine room fire data in real scenarios, and there is currently no dedicated public dataset for ship engine room fires. Considering that fires in different scenarios also have many similar characteristics, this paper uses the combination of fire images simulated in a 3D virtual engine room and captured in real scenarios to build a dataset. Images of real fires are downloaded directly from the Internet, and the simulated fire mainly relies on particle system modeling technology [43], which can build a flame model to simulate the fire occurrence process. Then, the simulated fire is integrated with the large 3D virtual engine room [44], so as to construct the fire in the engine room environment. The production process of fire simulation data is shown in Figure 11.

**Figure 12:**
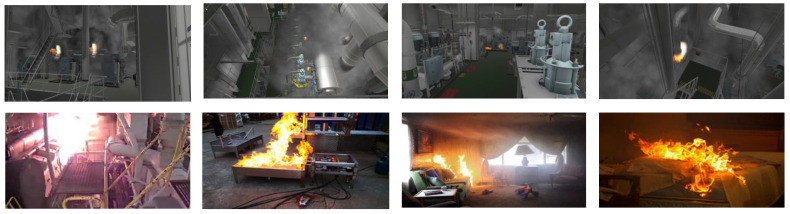
Examples of the dataset.

**Figure 13:**
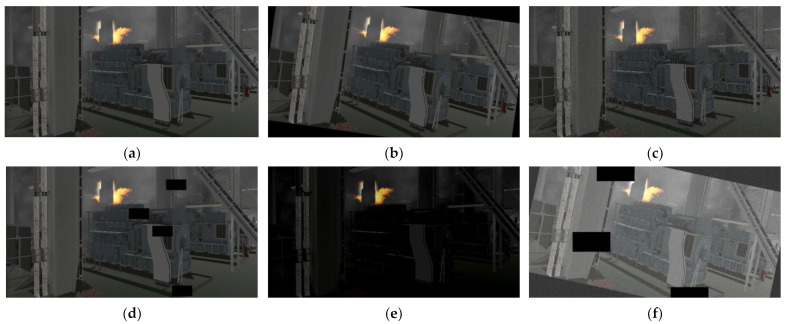
Examples of data augmentation. (**a**) Original image. (**b**) Rotation. (**c**) Noise. (**d**) Cutout. (**e**) Brightness. (**f**) Combination.

**Figure 14:**
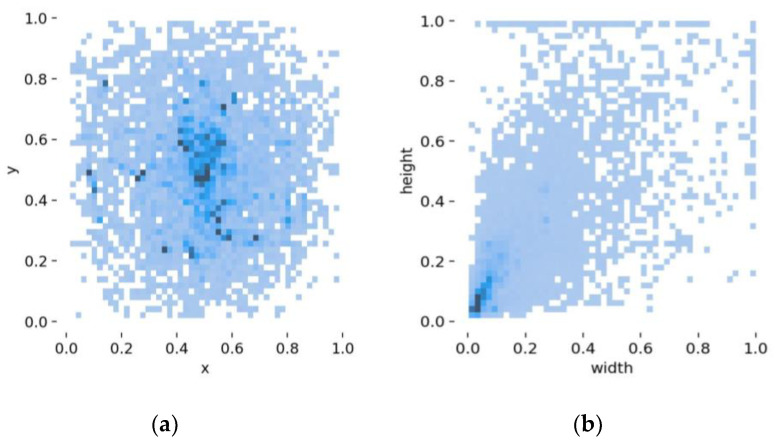
The visualization results of the analysis of the dataset. (**a**) the distribution of object centroid locations. (**b**) the distribution of object sizes.

In order to reflect the engine room fire as truly as possible, we selected the main engine area, the generator area, the oil separator area, and other places to collect the fire images from various angles and distances. Figure 12 shows some pictures in the dataset, in which the top half is some virtual cabin fire sample images, and the bottom half contains some real engine room fire pictures and flame pictures in other scenes. In addition, in order to enrich the diversity of the data and simulate the environment of the engine room, such as changes in lighting, we used data enhancement methods such as rotation, noise, cutout, brightness, etc. The data enhancement is shown in Figure 13. The final dataset of ship engine room fire consists of 12,986 images. We divided the dataset into a training set, validation set, and test set, according to the ratio of 6:2:2. Figure 14 shows the visualization results of the dataset analysis, where (a) represents the object centroid position distribution, the abscissa and ordinate represent the centroid position; and (b) represents the object size distribution, the abscissa, and ordinate represent the width and height of the object. It can be seen that there were more targets in the central region, and most of the targets were small targets, which is roughly in line with the actual situation. In addition, in order to make the test of the model more convincing, we downloaded a video of an engine room fire from the Internet as the test object. On the one hand, we can use this to simulate the detection of surveillance video models or on the other hand, it can also be used to test the generalization of our model, since the images in the video are all very new to the model.

### 4.2. Training Environment and Parameter Settings

The configuration information of our experimental platform is shown in Table 1. The operating system is Windows 11, the central processing unit (CPU) is Intel(R) Core(TM) i9-12900H, the graphics processing unit (GPU) is NVIDIA GeForce RTX 3070, random access memory (RAM) is 16 GB, and the code integration development environment (IDE) is PyCharm. The model is built based on the programming language Python 3.8.15 and the deep learning framework PyTorch 1.12.1. Finally, training acceleration, reasoning test, and verification are completed in CUDA 11.3.

The training parameter information is shown in Table 2. The input image size is 640 × 640, the batch size is 32, the momentum is 0.937, the initial learning rate is 0.01, and the weight decay is 0.0005.

### 4.3. Evaluation Metrics

Accuracy and speed are important metrics to measure the performance of the model. The metrics for detection accuracy include precision (P), recall (R), average precision (AP), and mean average precision (mAP). The speed of the model detection uses the frames per second (FPS). The calculation formulas are as follows:

(18) $$P=\frac{TP}{TP+FP}\times 100\%$$

(19) $$R=\frac{TP}{TP+FN}\times 100\%$$

(20) $$AP=\int_{0}^{1}P(R)dR$$

(21) $$mAP=\frac{1}{n}\sum_{i=1}^{n}AP_{i}$$

(22) $$FPS=\frac{N}{t}$$

where *TP* stands for correct detection, which means the model prediction is positive and the actual value is also positive. *FN* stands for detection error, which means the model prediction is negative and the actual value is positive. *FP* stands for the detection error, which means the model prediction is positive but the actual value is negative. TN stands for correct detection, which means the model prediction is negative and the actual value is also negative. n is the number of target categories, *AP_i_* is the *AP* of the target with serial number *i*, *N* is the number of images detected, and *t* is the time spent on detection. In addition, the volume of the model and the number of parameters are also used to analyze and verify the performance of the model.

### 4.4. Experimental Results and Analysis

#### 4.4.1. Comparison of Two Activation Functions

**Figure 15:**
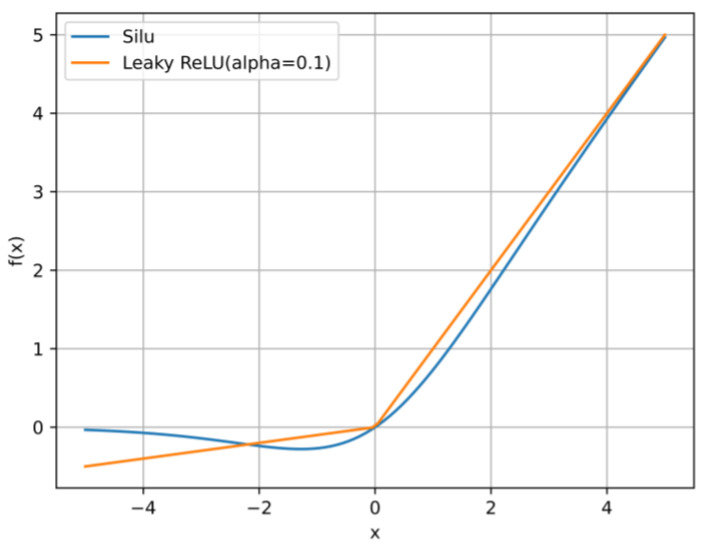
Graph of LeakyReLU and SiLU activation function.

First, we try to replace the LeakyReLu activation function with the SiLu activation function; both YOLOv5 and YOLOv7 use the SiLu activation function by default. Figure 15 shows their function curve. SiLu is also an improvement over ReLu, which has a smoother curve when approaching zero. The experimental results are shown in Table 3. Although SiLu is more advanced in theory, the actual performance of the experiment in this paper is mediocre. Therefore, LeakyReLu is still used as the activation function of YOLOv7-tiny model in subsequent experiments.

#### 4.4.2. Comparison of Different Backbone Networks

Then, we try to replace the backbone network of YOLOv7-tiny with several popular lightweight models, such as MobileNet, GhostNet, and ShuffleNet; they are compared with the improved model using PConv. All experiments used the same dataset and the same training parameters mentioned above, and the results are shown in Table 4. The experimental results show that these lightweight network models can significantly reduce the GFLOPs. However, with the decrease of GFLOPs, the model speed does not improve but decreases to different degrees. There is also a decrease in their mAP. Only the model using PConv achieves both speed and accuracy improvements.

#### 4.4.3. Comparison of Different Attention Mechanisms

In order to choose the appropriate attention mechanism, we added four attention mechanisms to the backbone network for comparison. The results are shown in Table 5.

**Figure 16:**
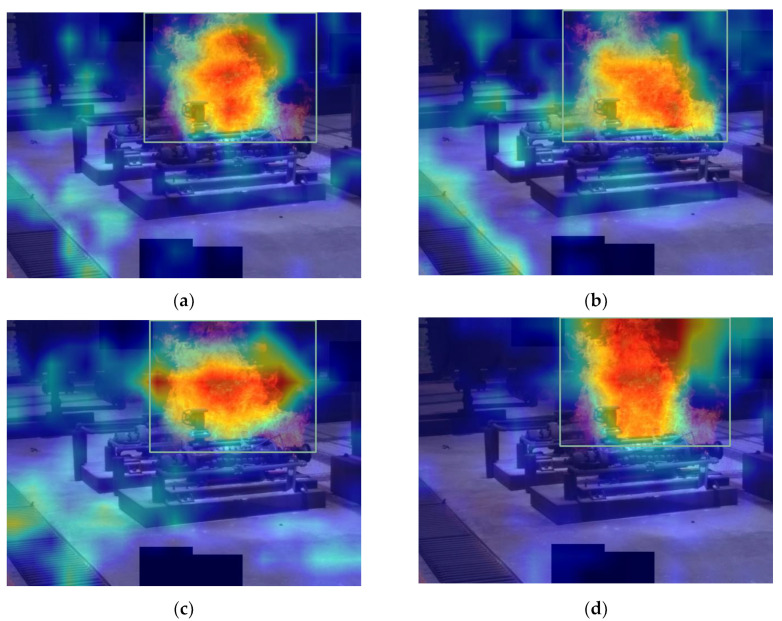
Grad-CAM flame heat map visualization results. (**a**) Heatmap of the network using ECA. (**b**) Heatmap of the network using CBAM. (**c**) Heatmap of the network using SE. (**d**) Heatmap of the network using CA.

Different attention mechanisms have different effects on the model, and they work differently depending on where they are added. After adding the attention mechanism to the backbone network and the head network, we find that the attention mechanism added to the backbone network can improve the performance of the model better. Compared with other attention mechanisms, the coordinate attention mechanism can consider both the channel dimension and spatial dimension, and can make the model pay more attention to useful channel information by learning adaptive channel weights. The Grad-CAM [45] method is used to compare the visualization results of the experiment and is shown in Figure 16. It can be seen that the Grad-CAM mask of the model with the coordinate attention mechanism can better cover the target. Its prediction range is more accurate, which also illustrates the superiority of the coordinate attention mechanism from the side.

#### 4.4.4. Comparison with The Original Network Model

**Figure 17:**
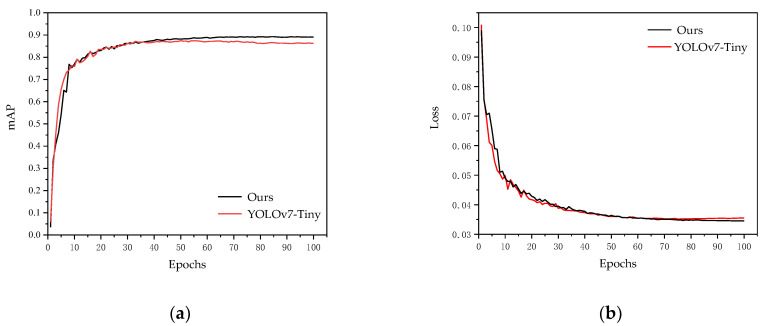
Comparison of validation set curves. (**a**) mAP of model validation set. (**b**) loss of model validation set.

In order to effectively analyze the performance of the improved model, the original YOLOv7-tiny network and the improved YOLOv7-tiny network were trained, respectively, by using the same data set and the same training parameters and training methods. The mAP and loss curve of the validation set during training are shown in Figure 17. It can be seen that the improved YOLOv7-tiny had higher mAP and lower loss on the verification set.

The performance of the model on the test set is shown in Table 6. Compared to the original YOLOv7-tiny, the volume, parameters, and floating-point operations of the improved model are significantly reduced. The mAP increased by 2.6%; The parameters were reduced by about 24%, and the amount of computation was reduced by about 27%. In addition, the model volume was reduced from 11.6 MB to 8.9 MB. The detection speed was increased from 96 frames per second to 106 frames per second, which can well meet the requirements of real-time detection and lightweight fire detection model requirements of embedded devices.

**Figure 18:**
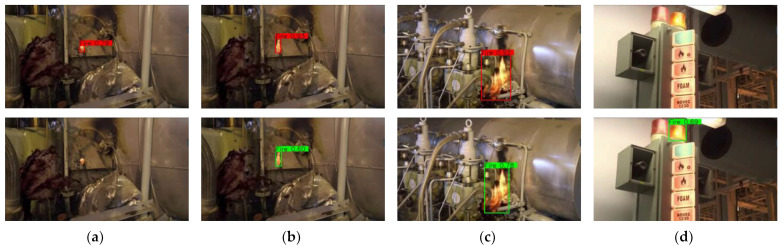
Video detection results. (**a**,**b**) small target flame. (**c**) medium target flame. (**d**) easily misjudged target.

In order to further validate and evaluate the improved model, we tested the original model and the improved model with a video of an engine room fire downloaded from the Internet. On the one hand, we can observe the detection effect of the model on the video. On the other hand, we can achieve the advantages of the improved model by comparison. Then we selected several representative frames of the video for presentation. Figure 18 shows the test results. The above is the test result of the improved model, and the below is the test result of the original model. (a) and (b) are small target flames representing the initial stage of the fire, which are more difficult to detect; (c) is a medium target flame; and (d) is a common alarm lamp post in the cabin, which is prone to cause misjudgment of the model. As shown in Figure 18, the original YOLOv7-tiny cannot detect a very small target, and the confidence level of the medium target is not high enough, plus there is also a misdetection. In contrast, our improved model avoids these problems. It can not only detect small targets to give an early fire alarm but better reduce misjudgments as well. The improvement of the fps and the detection effect of small target flames also shows that the model has the ability of real-time detection.

**Figure 19:**
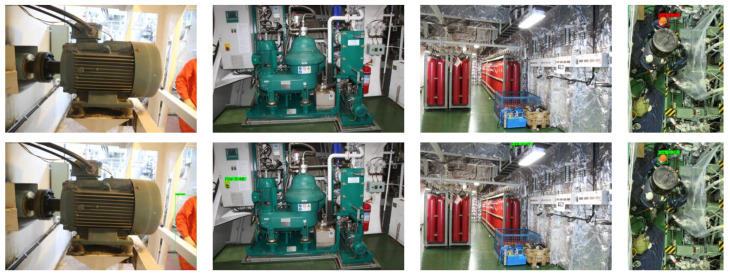
Easily misjudged object detection results.

The above tests show that our model has a good effect on video. In the engine room, however, there are many objects which may interfere with the model. We collected many pictures containing such objects to observe the performance of the model. These pictures include the crew in the engine room, kinds of machinery, signage, and so on. In order to show the results more clearly, we observe the performance of both models after setting the confidence level threshold to 0.2. They can detect most images successfully, but they also make misjudgments. Some of the results are shown in Figure 19. The top half is the improved model, and the bottom half is the original model. It can be obviously seen that after the improvement, the wrong judgment of the model reduced a lot, however, it still identified an orange safety helmet as a flame. Although we can set a high confidence threshold to reduce misjudgments, there is still room for improvement in our model in this aspect.

#### 4.4.5. Ablation Experiments

In order to analyze the effects of different methods, five groups of ablation experiments were designed. Each group of experiments used the same dataset and the same training parameters and methods to complete the training. The experimental results are shown in Table 7, where “-” means that the corresponding method is not used in the network, while “√” means that the method is used.

The first row in the table shows the result of the original YOLOv7-tiny network without any improvement measures. The effects of PConv and CA have been verified in previous chapters. According to the second and third rows of the tables, after adding only PConv and CA modules, respectively, mAP@0.5 increased by 1.6% and 0.4%, respectively. When replacing the CIOU loss function with the SIOU loss function alone, mAP@0.5 improved by 1%. It can also be seen from the data of other groups that these methods did not conflict when they were combined together. When these three methods were used together, mAP@0.5 achieved the highest result of 89%.

#### 4.4.6. Comparison with Others

Finally, we compare the improved model with other current mainstream detection models. The results are shown in Table 8. All models were trained using the same data set.

As observed in Table 8, our model has much higher speed and mAP compared to SSD and Faster R-CNN. Compared with other algorithms in the YOLO family, mAP@0.5 was 2.3%, 3.1%, 1.1%, 2.5%, and 2.1% higher than YOLOv3, YOLOv3-tiny, YOLOv4, YOLOv5n, and YOLOv5s, respectively. Although the speed was lower than that of YOLOv3-tiny, it was still enough to meet the requirements of real-time detection, and the gap in mAP@0.5 made up for it.

## 5. Conclusions and Discussions

In this paper, we propose a fire detection model based on an improved YOLOv7-tiny, which can be used to detect a ship engine room fire accurately and quickly. First, we use images of fire simulated in virtual engine room and captured in real scenarios to construct a dataset suitable for training the engine room fire detection models. Then, we introduce partial convolution and coordinate attention mechanisms to improve the detection speed and the feature extraction ability of the model. Finally, the original CIoU loss function is replaced with the SIoU loss function. The SIoU loss function introduces the concept of the angle between the true box and the prediction box to help calculate the distance between the two boxes and accelerate the convergence of the network. The results of experiments show that the mAP@0.5 of the improved model is 2.6% higher than the original. The speed of model detection is increased by 10 frames per second, reaching 106 fps, which meets the requirement of real-time detection. Compared with other detection models, ours is also more advantageous.

The model proposed in this paper can initially realize the fire detection of the ship engine room, but it still has the following shortcomings:

1. We only considered the flame in fire detection while ignoring the smoke. In fact, smoke often appears before the flames, so smoke detection is also very important for achieving fire early warning;
2. Some images in the dataset in this paper were obtained by simulation, which has some limitations;
3. We only researched whether there was a fire but did not consider the degree and development of the fire;
4. The research of this paper still stays in the algorithm mode without considering the practical application.

Therefore, we would like to mention the following aspects of our research, which may merit further study.

1. In terms of research objects, we will add the content of smoke detection. Using the existing conditions of the laboratory to make the dataset of smoke, so that the detection of fire is not limited to flames, which may make fire detection more scientific;
2. In terms of research content, we should not only consider whether there is a fire, but also carry out relevant research on the situation after the fire, such as the development of the fire, the escape path of the crew, and so on;
3. In terms of methods, we will combine more excellent and classical methods and algorithms to improve the model, such as long short-term memory (LSTM), which is often used in the field of natural language processing, or the temporal difference method in traditional moving object detection;
4. In terms of practical applications, although in theory we only need cameras and computers to complete the detection, we still need to conduct a more comprehensive study of the engine room environment and ship laws and regulations to evaluate its feasibility. The hardware devices, computing resources, and the device deployment also need to be considered;
5. In addition, it is better to detect the type of fire, such as A, B, or C fire, which can provide an indication of how to extinguish the fire.

## Figures and Tables

**Table 1:** The configuration information of the experimental platform.

Configuration	Versions
Operation system	Windows 11
CPU	Intel(R) Core(TM) i9-12900H @ 2.50 GHz
GPU	NVIDIA GeForce RTX 3070 Laptop GPU
RAM	16.0 GB
IDE	PyCharm 2022.1.2
Compiler	Python 3.8.15
Framework	PyTorch 1.12.1
Toolkit	CUDA 11.3 + cuDNN 8.2

**Table 2:** The training parameter information.

Image Size	Batch Size	Epochs	Momentum	Initial Learning Rate	Weight Decay
640 × 640	32	100	0.937	0.01	0.0005

**Table 3:** The result of the comparison of two activation functions.

Models	Precision	Recall	mAP@0.5	Speed (fps)
YOLOv7-tiny-LeakyReLu	89.2%	81.4%	86.4%	96.2
YOLOv7-tiny-SiLu	89.3%	83%	86.2%	92.6

**Table 4:** The comparison of different backbone networks.

Backbone	Precision	Recall	mAP@0.5	GFLOPs	Model Volume	Speed (fps)
MobileNet	88.7%	80.7%	84.7%	10.8	11.4 MB	86.9
GhostNet	90.4%	80.6%	84.9%	9.6	12.4 MB	77.0
ShuffleNet	88.1%	81.8%	85.7%	8.5	8.87 MB	79.4
PConv	88.6%	82.7%	88.0%	9.5	8.92 MB	102.0

**Table 5:** The comparison of different attention mechanisms.

Models	Precision	Recall	mAP@0.5
YOLOv7-tiny	89.2%	81.4%	86.4%
YOLOv7-tiny+CBAM	89.4%	82%	86.4%
YOLOv7-tiny+SE	89.2%	81.9%	86.7%
YOLOv7-tiny+ECA	89.2%	82.3%	86.3%
YOLOv7-tiny+CA	89.4%	82.2%	86.8%

**Table 6:** The results of original and improved YOLOv7-tiny.

Models	Precision	Recall	mAP@0.5	Parameters	Model Volume	GFLOPs	Speed
YOLOv7-tiny	89.2%	81.4%	86.4%	6,007,596	11.6 MB	13.0	96.2
Ours	88.9%	83.4%	89.0%	4,563,438	8.9 MB	9.5	106.4

**Table 7:** The results of the ablation experiment.

Model	Methods			Precision	Recall	mAP@0.5
	PConv	CA	SIoU			
YOLOv7-tiny	-	-	-	89.2%	81.4%	86.4%
	√	-	-	88.6%	82.7%	88.0%
	-	√	-	89.4%	82.2%	86.8%
	-	-	√	90.1%	81.4%	87.4%
	√	-	√	88.6%	82.2%	88.7%
	√	√	-	90.0%	81.2%	88.5%
	√	√	√	88.9%	83.4%	89.0%

**Table 8:** The results of other detection models.

Models	Input Size	mAP@0.5	Speed (fps)
SSD	300 × 300	70.8%	38.6
Faster R-CNN	600 × 600	80.1%	14.5
YOLOv3	640 × 640	86.7%	79.2
YOLOv3-tiny	640 × 640	85.9%	122.5
YOLOv4	640 × 640	87.9%	86.3
YOLOv5n	640 × 640	86.5%	94.1
YOLOv5s	640 × 640	86.9%	92.4
Ours	640 × 640	89.0%	106.4

## Data Availability

The dataset cannot be shared at this time, as the data also form part of an ongoing study.
